# Understanding the factors influencing implementation of a new national patient safety policy in England: Lessons from ‘learning from deaths’

**DOI:** 10.1177/13558196221096921

**Published:** 2022-05-06

**Authors:** Mirza Lalani, Sarah Morgan, Anamika Basu, Helen Hogan

**Affiliations:** Department of Health Services Research and Policy, 4906 London School of Hygiene and Tropical Medicine, London, UK

**Keywords:** patient safety, policy implementation, learning from deaths programme

## Abstract

**Objective:**

A new patient safety policy, ‘Learning from Deaths’ (LfD), was implemented in 2017 in National Health Service (NHS) organisations in England. This study examined how contextual factors influenced the implementation of LfD policy and the ability of the programme to achieve its goals.

**Methods:**

Semi-structured interviews were undertaken with key policymakers involved in the development of the policy, along with interviews with managers and senior clinicians in five NHS organisations responsible for implementing the policy at the local level. We also undertook non-participant observation of relevant meetings and documentary reviews of key organisation procedures and policies pertaining to LfD.

**Results:**

The study findings suggest several factors that hinder or support patient safety policy implementation at a local level. These include: (a) an organisation’s capacity and capability to support data collation, analysis and synthesis, (b) the dissemination of the resulting information, (c) the learning culture and hence perceptions of the purpose of LfD within an organisation, and (d) the extent of engagement in cross-organisational approaches to learning.

**Conclusions:**

Extra and intra-organisational contextual factors influence all stages of the policy implementation process from preparation and tracking to implementation support and review affecting its chances of success or failure. Successful adoption of a national patient safety policy within health care organisations can be informed by taking into consideration those factors.

## Introduction

The apparent success or failure of a policy is often determined by its implementation process. Factors shaping implementation are multifaceted, and policies created with linearity in mind are often subject to adaptation.^1^ Policy implementation comprises several key elements: policy preparation (characteristics, feasibility and practicalities of the proposals), policy tracking (monitoring progress), implementation support (managing and regulating, problem-solving and capacity building) and implementation review.^2^ Successful implementation is neither contingent on optimising all of the elements nor focussing on a single aspect but on the relevance and adaptability of each aspect.

The complexity of health care systems can impede effective policy design and implementation. In the United Kingdom, the National Health Service (NHS) and the Department of Health and Social Care have often responded to health care crises by introducing new national policy aimed at improving patient safety.^3^ As a result, NHS provider organisations find themselves grappling with multiple, often changing, patient safety priorities, which may not always be well aligned with local goals or activities. This may exacerbate the inherent tensions within policies and increase the potential for negative impacts when implemented into diverse health care organisations.^4^ Such policies are often not evaluated and so less is known about local implementation factors that hinder or enable a policy meet its intended goals.^5^ Local context is particularly influential, and aspects such as leadership, staff engagement and the availability of resources (especially local level expertise and skills) may largely determine the extent of adoption of a policy at a local level.^6^

In 2015, the publication of an independent review into Southern Health (a NHS provider of community health, specialist mental health and learning disability services) found that Southern Health investigated only 1% of unexpected deaths amongst patients with learning disabilities.^7^ A subsequent report published by the Care Quality Commission^8^ (the UK’s independent regulator of health and social care services) highlighted a fragmented approach to learning from deaths across the NHS. The concomitant findings from both reports opened a ‘policy window’^9(p264)^ facilitating the creation of a new policy framework, ‘Learning from Deaths’, published by the NHS National Quality Board in early 2017.^10^

The policy recommended the adoption of systematic case sampling and review of patient deaths, training of case record reviewers, and mechanisms for capturing and publicising findings and subsequent actions. The policy also contained an ambition that NHS organisations pursue wider investigation and learning across organisations and that approaches to dealing with families in the aftermath of deaths be improved. All acute, community and mental health NHS Trusts (the organisations that run most NHS hospitals) were required to have these processes in place by September 2017 and the adoption of the policy at a local level was to be monitored and assessed by the Care Quality Commission.^9^

To increase the speed with which the policy was implemented, it was wrapped around several initiatives already in place such as the Learning Disabilities Mortality Review Programme (LeDeR), the National Mortality Case Record Review (NMCRR) programme and the Duty of Candour which were developed to increase transparency, accountability and learning in the NHS.^9,10^

In this study, we examine how contextual factors such as patient safety priorities, governance structures, resource allocation and activities at the organisation level influenced the implementation of LfD policy and the ability of the programme to achieve its goals.

## Methods

### Data collection

The study was undertaken in two stages between July 2019 and August 2020. First, we conducted semi-structured interviews (*n* = 12) with policymakers. Using snowball sampling,^11^ the policymakers identified other individuals involved in the development of LfD. These interviews focussed on understanding the key drivers for the development of the policy, intended policy outcomes and likely challenges for implementation. The findings from these interviews have been published elsewhere.^9^

The second stage involved semi-structured interviews with managers and clinicians (*n* = 40) in five NHS Trusts. We purposively selected three acute Trusts and two community/mental health Trusts for participation based on their stage of adoption of LfD (early and later adopters). These Trusts covered district general hospitals, a large teaching hospital, and community and mental health providers.

Interviews were held in the participant’s place of work or using video conferencing, and lasted between 40 and 75 min. Each participant was provided a participant information sheet to obtain informed consent. There were no refusals to participate.

We attended several relevant meetings within the participating Trusts as non-participant observers and a member of the research team recorded field notes on meeting content. This was supplemented by documentary review of several site-specific documents associated with the Trust mortality programme. For more details, see online supplemental material S1.

### Data analysis

Interviews were audio-recorded and transcribed verbatim. Data were managed using NVivo version 12.0. We conducted qualitative analysis using a thematic framework approach to code the data and identify patterns and themes.^11^ The coding framework was developed using data from the stage 1 interviews with policymakers. It was from these interviews that we determined the five goals policymakers had for the LfD policy. These are detailed in Table 1.

**Table 1.** Intended goals of the LfD programme, as derived from our interviews with policymakers.

Goals of the LfD programme	Description
Establish a system to gather relevant information on deaths	Systematic approach to LfD, including an explicit case selection and review process that gathers both quantitative and qualitative information to identify any patient harm
Synthesise learning across the organisation	Integrating findings from LfD with other sources of safety information to maximise learning
Ensure organisation-wide learning and assurance, with transparent reporting on performance	Emphasis on examining deaths for learning, not just performance. Greater board involvement to raise the profile of quality and safety in the organisation and to support development of the Trust’s safety culture (accountability, transparency), reporting and learning
Improve experience for families	Better experience for families and carers, and mechanisms to gather valuable information from them to contribute to patient safety improvement
Promote inter-organisational learning across care boundaries	In cases where patients were cared for by several organisations across the local health and care system, establish systems whereby those organisations come together to examine the quality of that care

We mapped and categorised emerging themes from the phase 2 data against each of the five goals of the LfD programme. This enabled us to identify the key factors for implementation of the LfD policy (that is, the barriers and enablers to achieving the goals of the programme). Each researcher reviewed two to three transcripts from the Phase 2 data and themes were mapped against each goal. These were compared by the research team to create an initial coding framework which then progressed through several iterative stages until a final version of the framework was agreed upon. Around 50% of transcripts were coded by at least two of the researchers to enhance data validation. Findings from interviews were triangulated with meeting notes and document content. The research team held regular meetings to determine the categorisation of data under goals.

Our interviews and other data collection also enabled us to determine how the LfD process actually takes place at each Trust.

## Results

### Learning from deaths process

The mortality review process varied across the five Trusts, but generally case records of all deaths were screened by a senior clinician. In two of the acute Trusts, screening was undertaken by a Medical Examiner (ME). This is a senior doctor trained in the legal and clinical elements of death certification processes, who performs ME duties outside of their usual clinical work.^12^ Where quality of care concerns were raised at the screening stage, records would be sent for further in-depth reviews by a senior clinician usually not involved directly in the patient’s care. Other cases were also reviewed in depth if the death was judged to be unexpected (unanticipated or sudden) or conformed to LfD selection criteria.

Further clinical in-depth reviews of records used methods such as Structured Judgment Review (SJR).^13^ This blends traditional, clinical judgement with a standard format to examine the last episode of care prior to death. Subsequent learning from reviews was then shared at the Directorate and/or organisation level.

For more details, see online supplemental material S2.

### Goal 1: Establish a system to gather relevant information on deaths

Across all Trusts, the programme successfully promoted extensive adoption of systematic screening and review of deaths. Case notes of each death were screened for quality-of-care issues by a senior clinician. In the acute Trusts, at least 12% of deaths underwent a further in-depth review using the SJR process undertaken by a consultant not directly involved in the patient’s care. Most interviewees mentioned that screening and reviewing deaths was the most straightforward element of the LfD programme to adopt, acknowledging the widespread variability in performance in this area that existed previously.

It has given structure to a process which had previously been ad hoc. Different Trusts did it in different ways. But, even within Trusts, you found that different departments did things differently…If you want to have a decent governance process by which you can learn properly, you have to have people doing things the same way, or at least delivering information the same way. (Senior manager)

Interviewees remarked that the implementation of LfD was rapid and top down, limiting the time and effort taken by Trusts to adapt the programme into the organisation’s safety infrastructure, which, in some cases, resulted in parallel systems for delivery. Some interviewees remarked that the CQC, in tracking the policy, primarily assessed whether an organisation met the goals of LfD, with less concern about how it was implemented. Community Mental Health Trusts (CMHTs) identified LfD as better suited to acute Trusts. That was because they felt the policy has an emphasis on reviewing the last episode of care rather than the longer care trajectories typical of patients under their care. Acute Trusts with better performance on national bench-marking mortality indicators, such as the Hospital Standardised Mortality Ratio,^14^ also felt the programme was of less significance for them.

Many interviewees remarked that enthused and engaged programme leaders (mainly clinical) were instrumental in driving implementation. Clinical leaders provided a vision for integration of LfD at both strategic and operational levels, and promoted mainstreaming of the programme. Leaders were able to galvanise Board support and foster LfD alignment with other programmes with similar goals, such as the ME programme or incident reporting system. This proved important in leveraging internal resources both in terms of new money for extra posts, and for releasing consultants for SJRs or ME work. As one clinician said of the people who championed the programme at their Trust (referred to as ‘leads’):

The Learning from Deaths lead is both very capable and enthusiastic and the lead ME is again absolutely excellent and very committed to this initiative. You can always tell when you talk to people whether they believe in something or whether they’re going through the motions. When you have people in charge that feel that way, then that infiltrates the people they work with (Senior Clinician)

Interviewees reported that progress in the early stages of implementation was supported by peer collaboration facilitated by Academic Health Science Networks (organisations that connect NHS, academic organisations, local authorities, the third sector and industry). This promoted inter-organisational sharing of best practice and feedback on different approaches to implementation. One respondent recalled:

We’ve worked quite closely with [a neighbouring Trust], in terms of looking at the medical examiner system, and they shared some ideas about how to align the ME with learning from deaths. It’s been helpful because they were a pilot, they’re a well-oiled machine…It’s been quite good having them down the road to learn from. (Senior clinician)

### Goal 2: Synthesise learning across the organisation

Trusts were able to articulate clear benefits of the programme in improving end-of-life care and bereavement services. However, some interviewees believed that, overall, LfD was too reactive – problems were recognised but subsequent learning was limited to a single department or clinical team, with less consideration for how learning may be relevant across the organisation. Furthermore, measures put in place to prevent an issue from reoccurring were seldom assessed for impact. LfD also tended to draw more attention to relatively infrequent failures in care quality, which was seen as a barrier to staff learning:

I'm so supportive of [LfD]. But the fact [is], it came out of something that was fearful - that people were dying from needless deaths. And the focus initially was on what might have gone wrong, as opposed to all of the times that we get it right (Senior clinician)

Some interviewees suggested that their Trust had endeavoured to strike a balance, with learning from examples of good care, supplementing information from reviews of deaths with family and carer feedback. For example, one Trust produced ‘excellence’ reports from the collation of positive feedback from family members, which were shared with the Board resulting in teams being recognised for delivering good standards of care.

A prominent issue participants thought hindered the synthesis of organisational learning was a lack of integration of information from mortality reviews with other sources of patient safety data, such as serious incident reports or complaints. Interviewees identified two key barriers to the integration of information: (a) siloed information collation, analysis and learning and (b) limitations of IT systems to support data management. Trusts often relied on human integrators or committees (such as department-level safety committees) to collate information from different safety databases and sources. However, in many cases mortality data was siloed away from other sources of safety information. For example, in acute Trusts, findings from mortality reviews tended to be primarily reported to the Mortality Review Group whose primary focus was on LfD alone:

There is a bigger process than Learning from Deaths in the organisation. And Learning from Deaths does not feel like it assimilates into the wider process, it feels like it stands alone. Occasionally, we will come up against it and occasionally we’ll dovetail in neatly. (Senior manager)

Without access to broader information inputs, interviewees acknowledged that reviews of care quality issues remained narrowly scoped and over-focused on close antecedents to poor care providing restricted analysis of safety issues. This led to a sense amongst some clinicians and managers that there was limited value arising from the large numbers of mortality reviews being undertaken. In CMHTs, findings from mortality reviews were integrated into incident reporting pathways. This approach was one of the factors that supported the undertaking of broader thematic reviews or assessments of care pathways, combining data from LfD with other relevant quality-of-care data and enabling the focus to move beyond the last episode of care.

Trusts reported developing several mechanisms to overcome silos and facilitate LfD across the organisation. These included staff who could cross boundaries, such as Learning Disability Nurses, who were successful in transferring information between services (learning disability services and acute care). Furthermore, joint Mortality and Morbidity meetings, which bring together different specialities and professionals (such as junior doctors, nurses and physiotherapists) to LfD, were seen as an important forum to foster learning across different departments and clinical teams. The CMHTs’ thematic reviews were also an important way of engaging the wider multi-disciplinary team in learning together. Some Trusts had created a range of channels to communicate key messages from LfD to frontline staff from learning seminars and workshops to written materials.

### Goal 3: Ensure organisation-wide learning and assurance, with transparent reporting on performance

Some interviewees perceived the main purpose of LfD was assurance, regulation or performance management, despite assertions within the policy that the goal was learning and improvement. Participants attributed these views to the framing of the policy in the organisation and the nature of Board discussions around deaths, which varied across the Trusts. Participants at acute Trusts said that when mortality was discussed at Board meetings, the emphasis tended to be on its value as a bench-marking tool rather than for learning. Moreover, participants perceived that acute Trust Boards were particularly concerned that unfavourable comparisons could be made between organisations due to the annual LfD requirement to publish information from the programme. Participants in Trusts where the Non-Executive Director was actively engaged in LfD programme oversight said that this had resulted in more nuanced Board engagement, opening up possibilities to discuss safety from a broader perspective.

Interviewees also mentioned the potential for LfD to promote a more open culture among senior clinicians in particular. This could happen where senior clinicians believed leveraging combined elements of LfD and ME programmes could support a culture shift within Trusts, both by enhancing assurance, through independent scrutiny of case notes, and by improving transparency through creating more channels for open dialogue with families and carers. Participants reported that the ME had fostered an acceptance amongst consultants of the external scrutiny of their care, particularly when the process was accompanied by reassurance that the purpose was learning and improvement:

The initial process is when the consultants start getting asked all these questions, ‘Did you have any concerns?’ So, when they start getting the challenges, it’s probably a system they’ve not been used to. But I think most people know that if there’s a death, they will get a call. The team will get asked about the care of the patient, ‘Were there any concerns? Could anything have been done?’ (Middle manager)

### Goal 4: Improve experience for families

Broadly, many interviewees suggested that the LfD policy had resulted in improved communication and engagement with bereaved relatives. This was via MEs contacting family members and discussing their experience of care, including any concerns they may have. Additionally, personalised bereavement support – including the offer of additional conversations about death certification, possible coronial involvement or updates on progress over concerns previously raised with the ME – was regarded as an important step in reducing requests for reviews of death and promoting early concern resolution.

These improvements encouraged Trusts to develop open dialogue with families and to increasingly see families as partners in quality improvement with unique insights to offer:

I think [MEs] actually really felt the conversation with the bereaved, which wasn’t happening previously, and we have some sort of anecdotes and some attributes back from relatives about what that conversation felt and looked like, so you know, again some positivity around that. (Senior clinician)

However, participants acknowledged that more work needed to be done to involve families in incident investigation, to improve integration of family feedback with other sources of safety data and to provide prompt feedback to families once their concerns have been investigated.

### Goal 5: Promote inter-organisational learning across care boundaries

Interviewees from acute Trusts encountered challenges in engaging with cross-organisational LfD processes. This was because of a lack of time and resources, and difficulty involving key partners, such as GPs:

I have written to the Medical Directors and to the GPs to say Learning from Deaths would be much better if it was set within the system rather than within an organisation, because these cases are more complex than actually just meeting once or twice a year…[We could] look at two cases where it worked and two cases where it didn’t, and what can we learn as organisations. I’d find that much more useful than churning a hundred cases, which tell me it’s elderly people on end-of-life pathways. (Senior manager)

In contrast, CMHTs thought they had made good progress in cross-organisational LfD. This was due to their experience working with other providers, such as learning disabilities mortality reviews and vulnerable adult mortality reviews. Participants thought such forums encouraged organisations to share information, focussing on assessing care quality issues across a care pathway rather than within a single organisation.

## Discussion

This study has identified the key contextual barriers and enablers to implementation of LfD, providing insights into the contributory factors that may affect adoption of patient safety policies and their ability to meet key goals. The study findings suggest that our sample of NHS Trusts have identified approaches to partially address these contextual factors in order to promote adoption.

The design of a policy has implications for the way in which it is implemented. The LfD policy was rapidly formulated in response to a crisis, wrapped around several existing programmes with inherent tensions associated with its purpose (learning, transparency and accountability), and targeted all types of NHS Trust. Matland’s^15^ model of policy implementation recognises that adoption is influenced by the ambiguities and conflict in policy goals and their interpretation. Inevitably, LfD demonstrated ambiguity due to its multi-faceted ambition, but there was less conflict in relation to the focus on reviewing deaths given the broad consensus that learning from deaths was important and needed standardisation.

Engaged local programme leaders are key to facilitating policy adoption. They act as mediators engaged in knowledge transfer, assessments of readiness, capacity building, engaging senior clinicians and programme monitoring.^16^ We saw this in our study, with enthusiastic clinicians often vital to the successful adoption of LfD. The leaders’ roles included communication of a vision of how the new programme can be integrated with current safety priorities, securing internal resources, bringing clarity in terms of the roles and responsibilities at different levels in the organisation, and promoting ownership at the senior management level.

Internal and external cultural factors are potent influencers of any safety programme’s reception within a health care organisation.^17^ CMHTs were more likely to engage members of the wider multi-disciplinary team in LfD implementation, which has implications for both sustainability and wider dissemination of learning. Moreover, an inter-professional approach to learning, reaching beyond specialty boundaries, promotes the potential for transfer of multi-factorial and nuanced information about the quality of care across providers and services.^18^

In some Trusts, a Non-Executive Director with intimate knowledge of LfD could change the Board’s orientation and engagement in patient safety. This allowed for deeper understanding of safety issues and the opening up of broader perspectives while also placing the Board in a better position to fulfil its key role of providing scrutiny and constructive challenge.^19^ A focus on comparative performance and reputation in some Trusts orientated their LfD programmes towards predominantly identifying failures of care. Participants thought such an approach had a disengaging impact on clinical staff, enhancing fears of stigma and blame. There were also examples of learning from care that had gone well. Such learning, especially when delivered within current resource constraints is more likely to lead to sustainable change.^17,20^

Continued staff involvement in quality improvement programmes depends on demonstration of the benefits of the programme to achieving shared goals such as safer healthcare. This requires an effective means of disseminating learning across the organisation, rather than the data being siloed. LfD has seemingly bolstered efforts to improve end-of-life care and bereavement services, which had previously been identified as failing to reach key quality standards in many organisations.^21^ Yet, wider learning was inhibited by a propensity for reactive, single-loop learning and failure to close feedback loops.^22^ Broader approaches to learning from safety incidents and deaths, through thematic reviews or assessments of care pathways, moving beyond narrowly focused investigations of the last episode of care are more likely to identify whole-system problems and priorities for action. Likewise, participants commented that although staff were now better at gathering the perspective of bereaved families on quality of care, more needs to be done.

Finally, participants from acute Trusts identified a need for greater cross-institutional cooperation. For CMHTs this was a more natural approach, given the longer care trajectories of their service users and well-established cross-organisational relationships. Again, we see the role organisational culture plays in policy implementation.

Many of the external and internal contextual factors we identified are known key contributors to the successful implementation of a policy.^23^ The availability of resources, managerial and clinical leadership, engagement of a range of health care staff, and the culture within organisations are frequently cited as hindering or enabling implementation of quality and safety initiatives.^24^ As a consequence, policy implementation is said to be the product of policy, people and places,^25^ with the local context often the overriding determinant. Indeed, Dixon-Woods et al. suggest that quality and safety programmes are deemed to be working when a programme exhibits dynamic properties, such as the development of tailored versions of the programme to fit a local context.^26^

Our study provides some useful lessons that may have policy and practice implications for the implementation of patient safety policy in health care organisations. Policy preparation requires a clarity of purpose, with clearly articulated goals and limited scope for ambiguity and conflict. The elements of patient safety programmes most likely to survive are those that have become integrated into the local infrastructure and are seen as integral to delivery of strategic priorities for safety improvement. Tracking the policy by piloting it first could be used to illuminate potential issues before rollout. Implementation support that strengthens existing local level capacity and capability may address some of the contextual barriers identified in this study. Additionally, policy implementation review through ongoing monitoring and evaluation provides an understanding of programme evolution and the opportunity for adaption if intended goals are no longer being met.^27^

Furthermore, several current English NHS policy initiatives may contribute to addressing some of the challenges health care organisations face in implementing and integrating safety policies. Many of these are detailed in a new national patient safety strategy.^28^ For example, the introduction of Patient Safety Specialists as strategic leaders for safety in NHS organisations identifies a Trust individual with the remit to provide support for programme leads, facilitate Board backing for safety programme adoption and alignment and promote the shift to an open, learning culture.

Intelligent digital systems that allow the management of patient safety data to ensure its better use in supporting safety investigations are also required. These should have the capacity to enable user-friendly data entry, the undertaking of complex searches and data collation, and the broadening of access to data for a wider range of frontline staff.^29^ An ability to capture family and carer insights would be valuable. In-depth, broader focused safety investigations would provide greater opportunities for the identification of effective actions to improve patient safety. However, such an approach also requires health care organisations to prioritise those investigations judged most likely to have the greatest impact.

### Limitations

This study had one major limitation: It involved only a small sample of NHS organisations in England. Assessing the local level impact of the policy is challenging given the variation in implementation approaches across the surveyed Trusts and the difficulty in attributing change or improvement in organisation performance to a single patient safety policy. Nonetheless, some comparisons across the Trusts were made in terms of an organisation’s priorities, exemplified by the differing approaches of acute Trusts and CMHTs in the adoption of the policy.

## Conclusions

Given the complexity of health care systems and competing priorities, health care policy implementation is seldom straightforward. The organisations in this study have developed a number of approaches to leveraging the enablers and addressing the barriers that affect implementation. Their experience can inform the development of national patient safety agendas, increasing the likelihood of successful adoption of policy at a local level.

## Supplemental Material

Supplemental Material - Understanding the factors influencing implementation of a new national patient safety policy in England: Lessons from ‘learning from deaths’ Click here for additional data file. Supplemental Material for Understanding the factors influencing implementation of a new national patient safety policy in England: Lessons from ‘learning from deaths’ by Mirza Lalani, Sarah Morgan, Anamika Basu, and Helen Hogan in Journal of Health Services Research & Policy
