# Supplementary material for: Understanding the factors influencing implementation of a new national patient safety policy in England: Lessons from ‘learning from deaths’
Source: J Health Serv Res Policy. 2022 May 6;28(1):50–7. doi: 10.1177/13558196221096921 (PMC9850371; doi:10.1177/13558196221096921)
Supplement: Supplemental Material - Understanding the factors influencing implementation of a new national patient safety policy in England: Lessons from ‘learning from deaths’ [file sj-pdf-1-hsr-10.1177_13558196221096921.pdf]

Journal of Health Services Research & Policy
Understanding the factors influencing implementation of a new national patient safety policy in England: Lessons from Learning from Deaths
Lalani Mirza, Morgan S, Basu A, Hogan H

Supplement S1: Overview of data collection for stage 2

Research method	Participants/meetings/documents
Observation of meetings (30 hours)	Meetings included:<br>• Trust wide Mortality Review Groups (MRG) (*Multidisciplinary group that monitors and reviews information associated with the wider remit of mortality)*<br>• Serious incident meetings/panels<br>• End of Life care (EOLC) meetings<br>• Mortality and Morbidity Meetings (M&M) (*Meetings held primarily in hospital settings involving mainly doctors but also nurses and allied health professionals to learn lessons from clinical outcomes and drive service improvement)*<br>• Acute and Local Authority meetings involving registrars and coroners<br>• Academic Health Science Network (AHSN) regional mortality meetings
Interviews (n=40)	Non-executive Directors, Medical and Clinical Directors, Chief Medical Officers, Chief Nurses, Patient Safety Managers, Complaints and Incidents Managers, Medical Examiners and other senior clinicians such as consultants from different specialities
Documentary review	• Local LfD policy/guidance<br>• Quality Accounts (publically available report about the quality of services offered by an NHS healthcare provider)<br>• MRG meeting minutes<br>• Terms of reference for MRG<br>• Regularly drafted reports for Trust Board<br>• Annual Learning from Deaths reports

Supplementary S2: Summary of Learning from Deaths models at each Trust

**Trust type**	**Acute**		**Community**	
	Trust A and B	Trust C	Trust D	Trust E
**Screening**	Medical Examiners screen all notes of patients who died under the care of the Trust and hold discussions with bereaved family members about the quality of care received	No initial screen of cases where death occurred	Senior clinician screens case notes and categorises deaths as expected and unexpected	
**Referral (for further review)**	Deaths meeting LfD criteria or concern raised by family member		All expected deaths (unexpected - subject to Serious Incident Investigation)	Unexpected deaths
**Further review**	Structured Judgment Review - between 12-25% of deaths were selected based on LfD criteria as well as those deemed		Structured Judgement Review	In-depth review known as an initial review report or referred into

	useful for learning		Serious Incident Investigation
**Learning**	Directorate level M&M meetings<br><br>Trust wide-MRG	Learning from deaths panel (Trust wide)	Directorate MRG<br><br>Trust wide-MRG

Summary of learning from deaths criteria for referral for further review

**Learning from deaths referral for further review criteria:**
1. 'All deaths where bereaved families and carers, or staff, have raised a significant concern about the quality of care provision.'<br>2. 'All patient deaths of those with learning disabilities and severe mental illness.'<br>3. 'All deaths in a service specialty, particular diagnosis or treatment group where an 'alarm' has been raised with the provider through for example via a Summary Hospital-level Mortality Indicator, concerns raised by audit work, concerns raised by the CQC or another regulator.'<br>4. 'All deaths in areas where people are not expected to die, for example in relevant elective procedures.'<br>5. Deaths where learning will inform the provider's existing or planned improvement work.'<br>6. 'A further sample of other deaths that do not fit the 'identified categories' so that providers can take an overview of where learning and improvement is needed most overall.'
